# Nifedipine and Amlodipine Are Associated With Improved Mortality and Decreased Risk for Intubation and Mechanical Ventilation in Elderly Patients Hospitalized for COVID-19

**DOI:** 10.7759/cureus.8069

**Published:** 2020-05-12

**Authors:** Isaac Solaimanzadeh

**Affiliations:** 1 Internal Medicine, Interfaith Medical Center, Brooklyn, USA

**Keywords:** covid-2019, coronavirus disease (covid-19), pulmonary vasoconstriction, hypoxia, high altitude pulmonary edema, calcium channel blockers, nifedipine, amlodipine, pulmonary vasodilation, pulmonary artery hypertension

## Abstract

Dihydropyridine calcium channel blockers (CCB) are typically used agents in the clinical management of hypertension. Yet, they have also been utilized in the treatment of various pulmonary disorders with vasoconstriction. Severe acute respiratory syndrome coronavirus 2 (SARS-CoV-2) has been implicated in the development of vasoconstrictive, proinflammatory, and pro-oxidative effects.

A retrospective review was conducted on CCB use in hospitalized patients in search of any difference in outcomes related to specific endpoints: survival to discharge and progression of disease leading to intubation and mechanical ventilation. The electronic medical records for all patients that tested positive for SARS-CoV-2 that were at or above the age of 65 and that expired or survived to discharge from a community hospital in Brooklyn, NY, between the start of the public health crisis due to the viral disease up until April 13, 2020, were included.

Of the 77 patients that were identified, 18 survived until discharge and 59 expired. Seven patients from the expired group were excluded since they died within one day of presentation to the hospital. Five patients were excluded from the expired group since their age was above that of the eldest patient in the survival group (89 years old). With 65 patients left, 24 were found to have been administered either amlodipine or nifedipine (CCB group) and 41 were not (No-CCB group).

Patients treated with a CCB were significantly more likely to survive than those not treated with a CCB: 12 (50%) survived and 12 expired in the CCB group vs. six (14.6%) that survived and 35 (85.4%) that expired in the No-CCB treatment group (P<.01; p=0.0036). CCB patients were also significantly less likely to undergo intubation and mechanical ventilation. Only one patient (4.2%) was intubated in the CCB group whereas 16 (39.0%) were intubated in the No-CCB treatment group (P<.01; p=0.0026).

Nifedipine and amlodipine were found to be associated with significantly improved mortality and a decreased risk for intubation and mechanical ventilation in elderly patients hospitalized with COVID-19. Further clinical studies are warranted. Including either nifedipine or amlodipine in medication regimens for elderly patients with hypertension hospitalized for COVID-19 may be considered.

## Introduction

Nifedipine and amlodipine are dihydropyridine calcium channel blockers (CCBs) regularly used to treat hypertension. Yet, both medications have been utilized in the treatment of various pulmonary disorders with vasoconstriction as well. Severe acute respiratory syndrome coronavirus 2 (SARS-CoV-2) has been described to use the angiotensin-converting enzyme 2 (ACE2) receptor for entry into target cells expressed by the epithelial cells of the lung, leading to vasoconstrictive, proinflammatory, and pro-oxidative effects [1]. This vasoconstriction may play a role in the pathogenesis of the disease. Dysregulation or loss of hypoxic pulmonary vasoconstriction is suspected in Coronavirus Disease 2019 (COVID-19) as well [2-3].

A retrospective review of patients on either nifedipine or amlodipine was conducted in search of any difference in outcomes, including survival to discharge and progression of disease leading to intubation and mechanical ventilation. Patients in this population were prescribed either of these medications for the treatment of hypertension. Yet, reviewing outcomes in this context may reveal a benefit for the treatment of COVID-19 as well.

It is important to note the difference between dihydropyridine calcium channel blockers and non-dihydropyridines, as physiologic effects are likely not the same [4]. Whenever this article refers to a CCB, it is referring specifically and only to either nifedipine or amlodipine.

Background

Nifedipine was found to increase pulmonary vasodilation without decreasing arterial oxygenation or causing systemic hypotension in patients that suffer pulmonary hypertension from a chronic airflow limitation [5]. In tandem, amlodipine taken orally also produces acute pulmonary vasodilatation in patients with pulmonary hypertension [6]. Furthermore, amlodipine was also found to be an effective pulmonary vasodilator in patients with chronic obstructive pulmonary disease (COPD) with pulmonary hypertension [7]. In addition to being a safe and effective pulmonary vasodilator in these patients, it was also shown that amlodipine leads to an improvement in the right heart function [8].

During hypoxia, nifedipine significantly reduces pulmonary vascular resistance at both rest and exercise and inhibits hypoxic pulmonary vasoconstriction in patients with COPD [9]. In the same study, it was also found to substantially increase oxygen delivery during both rest and exercise.

Moreover, in patients with normal pulmonary artery pressures, nifedipine was shown to attenuate hypoxia-induced increases in pulmonary artery pressure and acutely dilates the constricted vascular bed associated with hypoxia in patients with COPD [10].

Although modulated via the endothelium, the core mechanism of hypoxic pulmonary vasoconstriction is in the smooth muscle cell [11]. The reversal of vasoconstriction with the use of dihydropyridine calcium channel blockers may be a method to improve outcomes in COVID-19. Nifedipine was previously observed to shift the pulmonary pressure-flow relationship to the right and increase the dispersion of blood flow distribution at rest and during exercise - strongly suggesting the release of hypoxic pulmonary vasoconstriction [12].

In light of this, the concomitant use of either nifedipine or amlodipine in patients hospitalized with COVID-19 was reviewed. Again, patients herein were treated with the calcium channel blockers for hypertension. Yet, this review sought to discover if a mortality benefit could be revealed in an acute illness requiring hospitalization for COVID-19.

## Materials and methods

A retrospective review of electronic medical records for all patients admitted to a community hospital who tested positive for SARS-CoV-2, who were at or above the age of 65, and who either expired or survived to discharge from hospital between the start of the public health crisis due to the viral disease (earliest admission date of a patient that tested positive at this hospital: February 27, 2020) and April 13, 2020. It is important to note that only patients with a final disposition on the day of study conclusion were included and that many more patients still hospitalized were not included in this review. The two groups were: (1) Treated with either nifedipine or amlodipine as part of the CCB group or (2) not treated with either amlodipine or nifedipine as part of the no-CCB group. Being “on” either of these medications required that they received more than one dose.

All patients in both groups were managed by a clinical team wherein antibiotics were administered in addition to hydroxychloroquine depending on patient consent and/or QTc prolongation status.

Patient outcomes were assessed for survival to discharge or signed out independently against medical advice (AMA) and expiration. Also looked at as a secondary outcome was the need for intubation and mechanical ventilation.

Clinical co-morbidities were reviewed in addition to demographic and clinical data.

Results were analyzed for statistical significance with the use of software available on these web pages: https://www.socscistatistics.com/tests/chisquare/default2.aspx and https://www.socscistatistics.com/tests/fisher/default2.aspx. The chi-square test and Fisher exact test calculator for a 2 x 2 contingency table were utilized for a statistical significance limit of P<.01 except where indicated.

To check for any significance between groups for factors that were continuous variables, the standard deviation was derived from the following website enlisted for statistical dispersion: http://www.alcula.com/calculators/statistics/dispersion/. Thereafter, the following website was utilized to calculate the comparison of means: https://www.medcalc.org/calc/comparison_of_means.php.

## Results

A total of 77 patients were identified. Of these, 18 survived until discharge and 59 expired. One patient signed out against medical advice (AMA) and this was included in the survival group. Seven patients from the expired group were excluded since they died within one day of presentation to hospital and the time frame of clinical deterioration limited potential therapeutic interventions. In order to attempt to age match the case and control groups, five patients were excluded from the expired group since their age was above the eldest patient in the survival group (89 years old). For the record, of these, only one was on a CCB and four were not on a CCB.

With 65 patients left, 41 were found to not have been treated with either nifedipine or amlodipine and 24 were found to have been prescribed and taking either nifedipine or amlodipine during the course of hospitalization. Demographic data are outlined in Table 1.

**Table 1:** Demographic Data and Comorbidities CCB = Dihydropyridine Calcium Channel Blockers (Nifedipine or Amlodipine). No-CCB = Not Having Taken More Than One Dose of Any Dihydropyridine Calcium Channel Blockers (Nifedipine or Amlodipine). NS = Not Significant.

Factor	CCB	Percent	No-CCB	Percent	P-value
Mean Age	74.91	(65-89)	75.59	(65-87)	NS
M	11	45.8%	21	51.2%	NS
F	13	54.2%	20	48.8%	NS
African American	19	79.2%	31	75.6%	NS
Other	5	20.8%	10	24.4%	NS
Hypertension	22	91.7%	34	82.9%	NS
Diabetes	15	62.5%	23	56.1%	NS
Bronchial Asthma or Chronic Obstructive Pulmonary Disease	5	20.8%	10	24.4%	NS
End-Stage Renal Disease	2	8.3%	4	9.8%	NS
Hyperlipidemia	2	8.3%	3	7.3%	NS
Anemia	2	8.3%	6	14.6%	NS
Congestive Heart Failure	2	8.3%	4	9.8%	NS
Benign Prostatic Hypertrophy	2	8.3%	5	12.2%	NS
History of Coronary Artery Bypass Graft	1	4.2%	2	4.9%	NS
Prediabetes	1	4.2%	2	4.9%	NS
History of Cancer	1	4.2%	4	9.8%	NS

In patients treated with a CCB, 12 (50%) survived and 12 expired, whereas only six (14.6%) survived and 35 (85.4%) expired in the No-CCB treatment group (P<.01; p=0.0036, see Table 2).

**Table 2:** CCB Medication Treatment Group vs. Survival Status CCB = Dihydropyridine Calcium Channel Blockers (Nifedipine or Amlodipine). No-CCB = Not Having Taken More Than One Dose of Any Dihydropyridine Calcium Channel Blockers (Nifedipine or Amlodipine).

	CCB	No-CCB
Survived to Discharge	12	6
Expired	12	35

**Figure 1:**
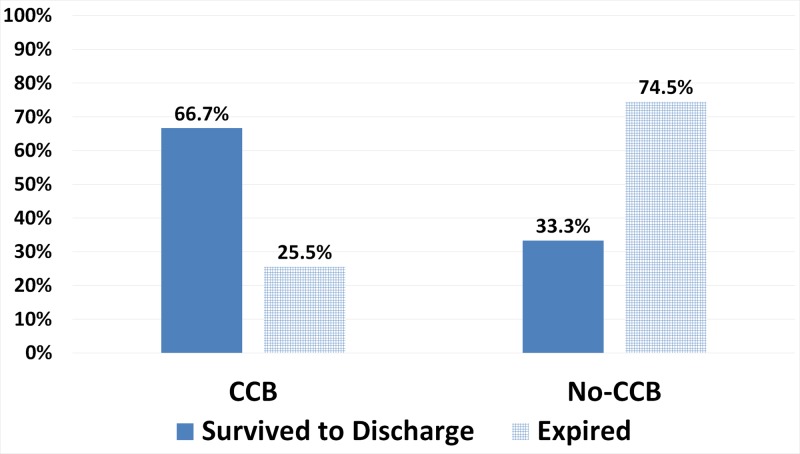
Percent Survival Vs. CCB and No-CCB Groups CCB = Calcium Channel Blocker (Nifedipine or Amlodipine). No-CCB = Not on Calcium Channel Blocker.

Considering this from a different perspective, 67% (12/18) of patients that survived and that were successfully discharged from the hospital were on a CCB, whereas 74% (35/47) of patients that expired were not on a CCB (Figure 1).

**Figure 2:**
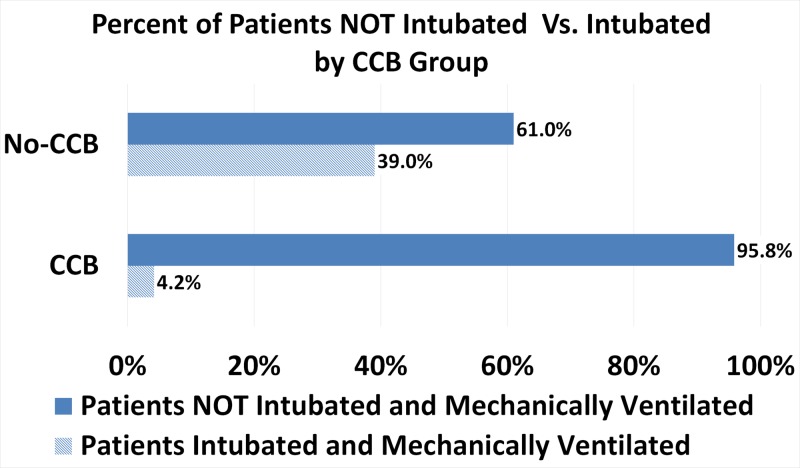
Percent of Patients Not Intubated by CCB Group CCB = Calcium Channel Blocker (Nifedipine or Amlodipine). No-CCB = No Calcium Channel Blocker.

Patients treated with a CCB were significantly less likely to have undergone intubation and mechanical ventilation. Since only one patient (4.2%) in the CCB group was intubated and mechanically ventilated and 23 (95.8%) were not, whereas 16 (39.0%) were intubated and mechanically ventilated and 25 (61.0%) were not in the No-CCB treatment group (P<.01; p=0.0026, see Table 3 and Figure 2).

**Table 3:** Patients Intubated and Mechanically Ventilated Vs. CCB Medication Treatment Group CCB = Dihydropyridine Calcium Channel Blockers (Nifedipine or Amlodipine). No-CCB = Not having taken more than one dose of any Dihydropyridine Calcium Channel Blockers (Nifedipine or Amlodipine).

	CCB	No-CCB
Number of Patients Intubated and Mechanically Ventilated	1	16
Number of Patients that were NOT Intubated and Mechanically Ventilated	23	25

Other sample medications administered were not significantly different between groups (see Table 4). Broad-spectrum antibiotics could be qualified with various medication regimens such as ceftriaxone in combination with azithromycin or doxycycline, vancomycin and meropenem, etc. Intravenous fluid comprised all patients that were administered at a minimum of 40 mL/hr at some point during the course of hospitalization. Steroid use included any type such as methylprednisolone, dexamethasone, or hydrocortisone.

**Table 4:** Miscellaneous Medications Between Groups NS = Not Significant. CCB = Calcium Channel Blocker (Nifedipine or Amlodipine). No-CCB = No Calcium Channel Blocker.

Intervention	CCB	%	No-CCB	%	P
Broad-Spectrum Antibiotics	24	100.0%	41	100.0%	NS
Intravenous Fluid	22	91.7%	33	80.5%	NS
Hydroxychloroquine	20	83.3%	32	78.0%	NS
Steroids	8	33.3%	12	29.3%	NS
Heparin	16	66.7%	25	61.0%	NS
Enoxaparin	2	8.3%	9	22.0%	NS
Venodyne Boots	2	8.3%	2	4.9%	NS
Apixaban	2	8.3%	1	2.4%	NS
Rivaroxaban	1	4.2%	3	7.3%	NS
Warfarin	1	4.2%	0	0.0%	NS
No Anti-Coagulation	0	0.0%	1	2.4%	NS

Several other factors, including vital signs and laboratory findings at initial presentation, were compared between groups as well (Table 5). Some patients did not have specific laboratory tests drawn; the number of patients included is indicated. Also, the first recorded pulse oximetry measure in many patients from both groups include levels obtained after immediate placement of oxygen supplementation was already initiated.

**Table 5:** Clinical Data Between Groups at Initial Presentation to Hospital * indicates significance at p<.05. N= Number of Patients. SD = Standard Deviation. BMI = Body Mass Index. Temp = Temperature. RR = Respiratory Rate. Sat = Pulse Oximeter Saturation. SBP = Systolic Blood Pressure. DBP = Diastolic Blood Pressure. MAP = Mean Arterial Pressure. Hb = Hemoglobin. GFR = Glomerular Filtration Rate. ESR = Erythrocyte Sedimentation Rate. LA = Lactic Acid. LDH = Lactate Dehydrogenase. CRP = C-reactive Protein. BNP = B-type Natriuretic Peptide. IL-6 = Interleukin 6.

	CCB	SD	N = 24	No-CCB	SD	N = 41	P value
BMI	29.0	±7.17	23	30.2	±7.45	40	0.5341
Temp	99.6	±1.91	24	98.8	±1.43	41	0.0652
Pulse	92.1	±16.79	24	100.8	±24.73	41	0.1326
RR	19.7	±2.06	24	21.3	±4.92	41	0.1347
Sat	94.0	±7.66	24	92.0	±8.26	41	0.3488
SBP	137.5	±27.08	24	124.6	±22.38	41	0.0421*
DBP	77.7	±12.05	24	72.4	±11.61	41	0.0861
MAP	97.6	±15.27	24	89.8	±13.54	41	0.0359*
Hb	12.5	±1.97	24	12.4	±2.54	41	0.8821
GFR	49.6	±28.49	24	36.4	±27.6	41	0.0700
ESR	62.9	±26.01	17	82.1	±28.24	21	0.0371*
D-Dimer	2559.1	±1783.93	17	5710.4	±5735.54	32	0.0328*
LA	1.9	±0.9741	12	4.0	±3.5623	31	0.0557
LDH	494.3	±195.12	17	725.2	±376.97	30	0.0234*
CRP	135.5	±76.67	15	175.7	±124.79	34	0.2546
BNP	252.1	±558.16	19	436.4	±836	32	0.3979
IL-6	77.4	±52.92	3	502.0	±944.77	9	0.2290

## Discussion

These results reveal that dihydropyridine calcium channel blocker (nifedipine or amlodipine) usage is associated with significantly improved mortality in elderly patients hospitalized for COVID-19. They also reveal that CCB usage is associated with a significantly decreased risk for intubation and mechanical ventilation.

This study reveals the possible benefits of nifedipine and amlodipine in patients hospitalized with COVID-19. Larger clinical studies are warranted. For those that already have hypertension and present to hospital with elevated blood pressure, assuming no contraindications exist, it may be fair to give preference to a CCB as first-line therapy for concomitant benefit. Further studies of other potential therapies that function as vasodilators in the pulmonary arterial flow should be pursued as well.

Clinical data comparisons between groups at the time of presentation were significant for differences in systolic as well as mean arterial blood pressure. This may indicate higher levels of hypotension or at least a lack of elevated blood pressure measures in the No-CCB group at presentation and/or foreshadow treatment with a CCB anti-hypertensive medication. Differences in erythrocyte sedimentation rate, D-dimer, and lactate dehydrogenase may reflect a disparate severity of disease upon presentation. However, on the other hand, this reasoning may be countered since results between levels of lactic acid, C-reactive protein, B-type natriuretic peptide, and interleukin-6 were not significantly different, in addition to no significant difference between hemoglobin and glomerular filtration rate (GFR).

It is not known if patients were taking a CCB at home or not. This may be another avenue to explore with consideration to decipher if the severity of disease expression is diminished or halted by CCB medication - prior to virus exposure.

Future studies may investigate different patient populations. Moreover, CCB treatment, while monitoring blood pressure closely in patients that are suffering from COVID-19, and that do not have underlying Hypertension, may be envisaged. Blood pressure is generally not hypotensive in many hospitalized patients suffering from COVID -19 and can even be higher in patients in the intensive care unit (ICU) [13].

The limitations of this study may be inherent in the small sample size, possible confounding factors not otherwise accounted for, and even selection bias as part of prior clinical decision-making to treat hypertension with a CCB or not. Therefore, larger and more rigorous studies should be pursued. Prospective studies may be considered as well. Large health systems may be in a unique position to help advance knowledge on this subject matter by conducting a more robust retrospective review. If clinical researchers therein would peruse electronic medical records (EMR) for similar outcomes, as described in this paper, that can aid in gaining a better understanding of the role that CCBs may have in mitigating disease. Particular attention may be paid, where possible, on patient adherence to outpatient medication regimens prior to acute hospitalization. For example, if the rates of hospitalization for patients that were adherent to CCBs were decreased, that might indicate preventative benefits. This could potentially explain significant differences of clinical data at the initial presentation described above, but this remains to be investigated. With the current EMR technology available at some institutions, this may be investigated rapidly. Results obtained may yield benefits for thousands of patients across the country and throughout the world that have not yet fallen ill but remain at risk for contracting this disease.

The need for mechanical ventilation likely represents the continuum of progression of the disease. It can be regarded as an intervention aimed at curtailing a trajectory towards mortality. However, mechanical ventilation should not be considered an all-encompassing treatment option for patients with COVID-19. In a recent study, amongst 1151 patients intubated, only 38 (3.3%) were discharged alive, with 24.5% that died and over 70% still in hospital [14]. Avoiding intubation with the utilization of potential countermeasures, such as with CCBs and other vasodilators described below, can be considered and evaluated since they may aid in the achievement of improved outcomes. When precisely during the course of the illness (early vs. late) these medications may be most effective may also be examined. Furthermore, perhaps beneficial effects of CCBs and other vasodilators described below can be extended to patients that are already mechanically ventilated. Clinical studies may investigate if successful weaning from mechanical ventilation is promoted by the use of vasodilators. Therefore, it is incumbent upon the medical community to exert their best efforts, on behalf of the many patients at risk, and pursue further evaluation as part of efforts to enhance clinical interventions that compel augmentation.

Concept

This data provides an impetus to explore different approaches to the treatment of patients with COVID-19. Focusing on vasodilatory agents may allow for an alternative treatment strategy. Herein, improved flow via the alveolar-capillary unit may be achieved. With improved flow, impediments to oxygenation, including inflammation and vasoconstriction, may be better negotiated. Furthermore, blood transit improvement as a result of vasodilation may potentially offset clot formation. Lastly, fluid accumulation or edema that can inhibit oxygenation may be collectively reduced as well. Altogether, the improved flow may attenuate the precipitous progression of the disease.

Virchow’s triad highlights three aspects compromising blood flow: stasis, hypercoagulability, and endothelial injury. All three may be occurring in advanced COVID-19. Yet, the progression to severe disease consisting of an inability to oxygenate may be the endpoint of a gradual process. In other words, flow (or micro-perfusion) via the alveolar-capillary unit may be slowly but surely decreasing as a result of a vicious cycle wherein inflammation secondary to viral injury begets hypercoagulability as well as the impedance of blood flow. Clot formation certainly lulls or wholly undermines segments of previously oxygenating pathways passing through the alveolar-capillary unit. Moreover, inflammation by itself, enhanced by the recruitment of cytokines, leukocytes, and the whole gamut of caustic endogenous mechanisms, may further render viable tissue non-functional. Additionally, with inflammation comes fluid or edema formation - this also compromises oxygen diffusion. On top of all this exists the innate disposition or tendency for hypoxic pulmonary vasoconstriction [15]. Perhaps, vascular inflammation also contributes to elicit reactive vasoconstriction independently. Altogether, a microvascular process may be occurring over numerous alveolar-capillary units, which yields a macro result. In sum, with increasing hypoxia and respiratory failure, the following challenges are faced and each promotes the other perhaps in sequence but not necessarily: (1) Viral injury provoking inflammation, (2) recruitment of an Immune response, (3) fluid accumulation, (4) vasoconstriction or compromised vascular flow, and (5) hypercoagulability and clot formation.

Multi-faceted challenges are faced in various clinical cases. However, the ultimate development of clot formation may not be applicable to many, if not most, patients upon presentation. These patients must be distinguished as not being in a category wherein the precipitous decline just described has already been realized. This is especially early on in the illness, since some may be managed, improve, and recover with supplemental oxygen and conservative fluid management or gentle fluid restriction alone. Herein, a vasodilator can be utilized from the outset since inflammation has not been prolonged, whereby flow via the alveolar-arterial complex is still consistent and clot formation likely has not already developed. However, in patients that have had prolonged symptoms, a consistent deterioration of oxygen saturation and/or hypoxemia should be evaluated with the consciousness that all five challenges may have already been established and taken form. Other patients maybe somewhere in between.

Broader implications

This being said, consideration of other vasodilatory agents should be pursued. These may include phosphodiesterase inhibitors sildenafil and tadalafil, as well as acetazolamide among others.

For example, sildenafil was shown to increase exercise capacity during severe hypoxia, as well as reduce hypoxic pulmonary hypertension at rest and during exercise while maintaining gas exchange and systemic blood pressure [16]. The same study also revealed that it yields an increased maximum workload and maximum cardiac output compared with the placebo.

Beyond this, phosphodiesterase inhibitors have the added benefit of improved renal perfusion and GFR - a valuable commodity as kidney disease is associated with the in-hospital death of patients with COVID-19 [17,18]. Also, when not administered with nitrates, sildenafil use resulting in hypotension, orthostatic hypotension, and syncope were found to be less than 2% [19-20]. Tadalafil once daily was found to improved exercise capacity and reduced time to clinical worsening in patients suffering from pulmonary arterial hypertension (PAH); offering an alternative to Sildenafil as well [21]. Finally, combining tadalafil with acetazolamide, rather than taking acetazolamide alone, can be an even more effective method for the prevention of some conditions [22]. Dosing of sildenafil is less restrictive in cases of compromised renal function.

Acetazolamide also attenuates hypoxic pulmonary vasoconstriction but has the added benefit of increasing minute ventilation and oxygenation [23-25].

Acetazolamide, however, requires close monitoring of arterial blood gases, prior to and following use, as treatment is contraindicated in metabolic acidosis; a condition that it can spur. However, some of the adverse effects of acetazolamide can be avoided by reducing the dose to compensate for age‐related reductions in renal drug clearance [26]. In any case, the addition of sodium bicarbonate can be utilized to counteract an acid tide and may be administered repeatedly in an alternating fashion with acetazolamide [27]. Acetazolamide also acts to inhibit carbonic anhydrase in vascular smooth muscle and this mechanism may be achieved by means of pH changes therein [28]. Clinical status and work of breathing must also be monitored closely. Patients that are already on a ventilator may also stand to benefit most from acetazolamide, as the control of various parameters may be adjusted for the optimization of therapy. An additional asset of acetazolamide includes diuresis of fluids - many, if not most, patients have significant crackles present on auscultation, and this likely hinders oxygenation as well (the latter is a clinical observation) [29].

Thus, acetazolamide can provide a triple benefit: diuresis of fluid/pulmonary edema, improved ventilation, and reversal of pulmonary vasoconstriction.

There is a caveat to all of this. That is, improvement of oxygenation and ventilation can only be pursued if clot formation does not exist or is previously adequately treated. A recent study found that an incidence of thrombotic complications is up to 31% of ICU patients with COVID-19 [30]. This must be addressed as well.

For example, in patients that are early stage and without markedly elevated D-dimers, preferably younger and not elderly, wherein crackles are grossly apparent on auscultation, the use of a vasodilator such as acetazolamide or a CCB may potentially stave off intubation independently. However, in an elderly patient, with markedly elevated D-dimers and little to no crackles with clear air movement on auscultation, it may be ineffective as alveolar-capillary units that are already clotted may harbor a formidable barrier towards improvement. Therefore, treatment with anti-coagulation prior to vasodilator therapy should be considered in these patients. Treating any clots or microclots first may allow for the effective flow once vasodilator therapy is implemented.

Context

The vasodilatory agents mentioned in this article may enhance clinical outcomes in patients suffering from COVID-19. Yet, they should be accompanied with considerations for anti-coagulation and anti-inflammatory agents as well, not to mention antibiotics for the prevention of co-infection and anti-virals that may also contribute towards resolution.

Since adding a vasodilatory agent in a patient that has had prolonged disease and may have already developed a clot or microclots may not suffice. Anti-coagulation, whether in prophylactic or treatment doses, may aid in reducing clot formation or extension. Therefore, combining regimens particularly in elderly patients and/or those that have had a prolonged course may be prudent. If anti-coagulation is administered, certainly close monitoring for any signs of bleeding must be implemented. Nonetheless, some patients may recover without anti-coagulation as well, and clinical acumen is necessary in all circumstances.

Fluids are an important subject matter that must be appreciated within the context of overall management, although it certainly deserves further assessment beyond this article. Generally, in patients that have stable blood pressure, no significant clinical signs of dehydration, and crackles on auscultation, conservative fluid management seems to be best. Ground-glass opacities present on imaging may be reflective of fluid collection as well. In these cases, avoiding intravenous fluid hydration and relegating to oral fluid intake as needed may decrease the risk of excess fluid accrual in the lungs. This approach is not feasible in many clinical situations such as where co-morbid diabetic ketoacidosis, severe dehydration, hypernatremia, or Rhabdomyolysis occurs. Nonetheless, oxygenation impedance as a result of edema formation is still worth taking into account in clinical management over an extended course of hospitalization. On the other hand, patients that are dehydrated from the outset or were previously on anti-coagulation prior to presentation and perhaps taking a CCB may have relatively clear sounding lungs on auscultation - this is a clinical observation. Those previously taking anti-coagulation and/or a CCB might possibly be less prone to fluid accumulation given preemptive counters to the impedance of flow and clot formation. This may be analogous to a pipe wherein, if flow is preserved, fluid may pass without interference. However, if it is clogged then certainly running more water will rebound and not successfully traverse the pipe without backing up or collecting proximally. In the former, where significant interference is not present, fluid may be a boon to expedite the clearance of inflammation. In the latter, where viral triggers of inflammation, down the five-step theoretical pathogenesis described above, have already exerted influence, it may worsen clinical status. The "pipe" analogy should be further qualified since, in our case, vascular channels may not be intact, as endothelial injury and capillary permeability are both likely. Moreover, inflammation in itself is prone to fluid accumulation as seen in patients with bowel wall edema following surgery, ascites in cancer, or serositis. Yet, while abdominal fluid accumulation may be a cause for significant discomfort, even mild pulmonary edema development may swiftly compromise oxygenation.

Finally, the use of steroids in severe cases and potentially in all hospitalized patients may aid in alleviating inflammation but, certainly, this is under investigation. With the concomitant use of vasodilators and anti-coagulation, the benefits of steroid use in COVID-19 may become more apparent.

All in all, in severe cases, early utilization of one or more vasodilator agent(s), anti-coagulation, steroids, and a diuretic (if no contraindications exist, preferably acetazolamide, given the additional benefits mentioned above), in addition to antibiotics and potential antivirals may be one protocol pathway to consider (Table 6).

**Table 6:** Sample Potential Example of a Therapeutic Approach That Can Be Considered for Evaluation *Consider 125-250 mg q6hr IV in mechanically ventilated patients combined with sodium bicarbonate 50 mEq q12hr to offset acid tide; attempt weaning from the ventilator in parallel with treatment. **Consider in patients with acute kidney injury. mg = Milligrams. mEq = Milliequivalent. kg = Kilogram. IV = Intravenous.

Vasodilator(s)	AND/ OR	Vasodilator-Diuretic	AND	Anti-Coagulation	AND	Steroid	AND	Antibiotics	AND	Anti-Viral
Nifedipine Extended-Release 30-90mg Daily OR Amlodipine 5-10mg PO Daily		Acetazolamide* 125mg PO or IV q8-12hr		Enoxaparin 1mg/kg q12-q24 hours		Methylprednisone 80mg IV then 40mg q12 hours		Ceftriaxone and azithromycin or doxycycline		Anti-viral
AND/OR										
Sildenafil** 20mg PO then 20-50mg Q8 hours or 10mg IV q8-12 hr										

Therapeutic interventions herein reflect the five-step progressive course outlined above. Patients that are otherwise stable but requiring oxygen supplementation to maintain a stable saturation level may improve with a vasodilator such as nifedipine or amlodipine (maximizing the dose to achieve ideal blood pressure may be of interest), prophylactic anti-coagulation, steroids, antibiotics and antiviral therapy alone without the use of a diuretic agent. Yet, if the clinical disease progresses and suspicion of need for invasive ventilation emerges, a diuretic such as acetazolamide may be considered. In ventilated patients, acetazolamide may be a key to promote weaning as described above. Frequent and repeated doses of acetazolamide may be necessary since the inflammatory effects of the viral provocation, as well as capillary permeability, will not vanish instantaneously and following a brief course of treatment re-accumulation of fluid may materialize. Therefore, consistent perseverance may be a successful strategy to quell the buildup of fluid, maintain vasodilation, and allow for conquest over time. Indeed, all vasodilation agents may exert their benefit over a gradual time course. Just like the decline of patients succumbing to COVID-19 occurs over an extended time course of worsening pathogenesis, so too the vasodilation agents may mitigate the same pathogenic process over an extended course of the viral illness. In other words, treating for just two days may not suffice even though some clinical improvement is observed. Stopping at that point may incur a rebound in status. Rather, a course of treatment at least five to seven days and up to two to three weeks in severe cases may be best.

It is worth noting that a relatively minor subset of patients, tending to be elderly and frail, has been observed to have disproportionately cold hands and feet relative to their extremities and the rest of their body. Additionally, they may be markedly sleepy and difficult to arouse. A pulse oximeter may fail to retrieve a saturation level when placed on their fingers and may yield a result only after being placed on their forehead. This may reflect an underlying lack of perfusion to extremities and conserving mechanisms for blood distribution. These patients are extremely ill and, although they may have stable vital signs, may be at risk for rapid deterioration. Similar treatment may ensue, but with compromised oral intake, intravenous fluid hydration may be needed; this may be best conducted at a gentle, gradual, and consistent rate. This subgroup should be recognized as possibly being part of a separate group of patients with an advanced illness that may require special attention.

Any clinician that has encountered patient presentations on a COVID unit, ICU, or emergency department is aware that presentations of COVID-19 are disparate and each individual case is different. Nonetheless, while correcting any concomitant disturbance (e.g. renal failure, hyperosmolar syndrome, hypernatremia, etc.), bearing in mind the central role of pulmonary deterioration in overall clinical demise is crucial. Ultimately, morbidity and mortality in COVID-19 may be a result of a failure to accommodate the pathogenic sequelae of toxic viral provocation rather than an immunologic deficiency. Therefore, aiding in the adaptation and negotiation of a physiologic response to the transient viral insult may effectively mitigate disease burden and promote improved outcomes. Altogether, implementing a strategy that optimizes flow via the alveolar-capillary unit may be a progressive path forward.

## Conclusions

In this small retrospective review, dihydropyridine CCBs were found to be significantly associated with improved mortality, as well as a decreased risk for intubation and mechanical ventilation in elderly patients hospitalized with COVID-19. Larger clinical studies are warranted. Future studies may also elucidate results in different patient populations and possibly reveal benefits even in mechanically ventilated patients. Consideration of treatment with a CCB in patients who are suffering from COVID-19 and that do not have underlying hypertension may be studied as well. Other potential therapies that function as vasodilators in pulmonary arterial flow should be pursued. Potential benefits may outweigh the risks of including nifedipine or amlodipine in the treatment regimens of elderly patients with hypertension hospitalized with COVID-19.
